# Supplementary material for: Dynamics underlie the drug recognition mechanism by the efflux transporter EmrE
Source: Nat Commun. 2024 May 28;15:4537. doi: 10.1038/s41467-024-48803-2 (PMC11133458; doi:10.1038/s41467-024-48803-2)
Supplement: Supplementary file 1 — Supplementary Information [file 41467_2024_48803_MOESM1_ESM.pdf]

Supplementary Information

Dynamics Underlie the Drug Recognition Mechanism by the Efflux Transporter EmrE

Jianping Li[1+], Ampon Sae Her[1+], Alida Besch[1+], Belen Ramirez-Cordero[1#], Maureen Crames[1#], James R. Banigan[1], Casey Mueller[1], William M. Marsiglia[1], Yingkai Zhang[1,2], Nathaniel J. Traaseth[1*]

[1] Department of Chemistry, New York University, New York, NY, USA

[2] Simons Center for Computational Physical Chemistry, New York University, New York, NY, USA

[+] These authors contributed equally to this work

[#] These authors contributed equally to this work

* Corresponding author: traaseth@nyu.edu

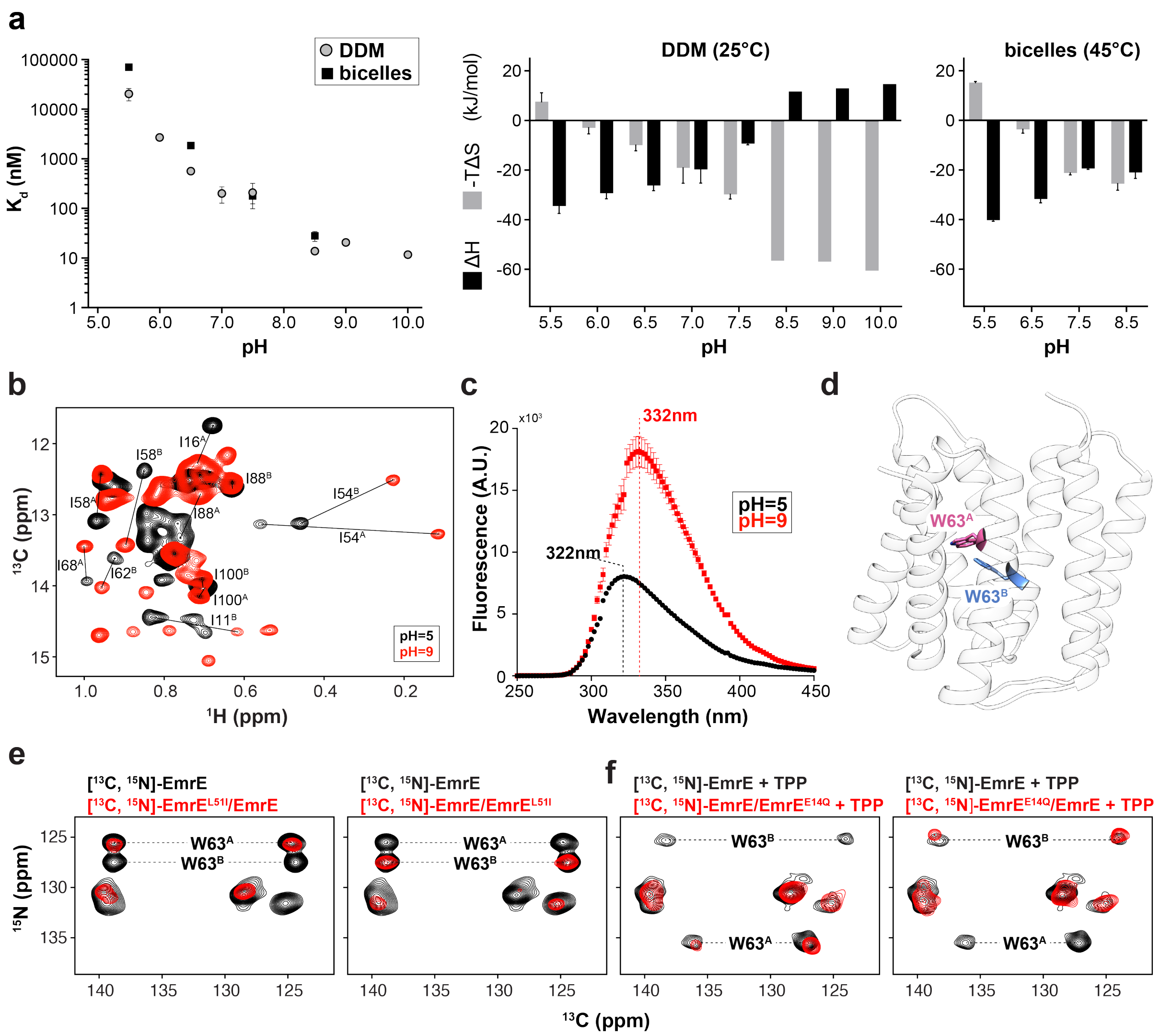

Supplementary Figure 1. EmrE structural changes are induced by pH and correlate to altered binding affinity to TPP.

a. Left: TPP binding affinities to EmrE derived from ITC experiments as a function of pH in DDM detergent micelles at 25 °C (grey circles) and DMPC/DHPC isotropic bicelles at 45 °C (black squares). Center and right: enthalpic (black) and entropic (grey) components of binding from ITC experiments determined in DDM detergent micelles (left) and DMPC/DHPC isotropic bicelles (right). Data in DDM micelles are presented as mean values ± s.d. for experiments performed more than two independent times (i.e., pH values < 8.5); binding data at pH values of 8.5, 9.0, and 10.0 were performed two independent times and the mean of these

experiments are displayed with no error bars. Data obtained in isotropic bicelles were replotted from Thomas *et al.*[1] and Shcherbakov *et al.*[2].

b. $^{1}H/^{13}C$ HMQC spectra of ^{13}C-Ile methyl labeled EmrE at pH 5 (black) and pH 9 (red) in DMPC/DHPC isotropic bicelles at 15 °C. Peak labels indicate the assigned isoleucine residue with the monomer in superscript.

c. Tryptophan fluorescence spectra of an EmrE mutant with a single Trp63 (W31F/W45F/W76F) in DMPC/DHPC isotropic bicelles at 25 °C. Spectra in black circles and red squares correspond to data collected at pH 5 and pH 9, respectively. Data are presented as mean values ± s.d. among three replicates from one independent experiment. The same trend was observed in other independent experiments.

d. The location of the two Trp63 residues highlighted in the NMR structure of proton-bound EmrE. Superscripts "A" or "B" refer to the corresponding monomer in EmrE.

e. Overlays of $^{13}C/^{15}N$ MAS correlation spectra of [^{13}C, ^{15}N]-EmrE (black) and the heterodimers [^{13}C, ^{15}N]-EmrE^{L51I}/EmrE (red, left panel) or [^{13}C, ^{15}N]-EmrE/EmrE^{L51I} (red, right panel) in DMPC lipid bilayers at pH 5.0. Superscripts "A" or "B" refer to the corresponding monomer A or B, respectively.

f. Overlays of $^{13}C/^{15}N$ MAS correlation spectra of [^{13}C, ^{15}N]-EmrE (black) and the heterodimers [^{13}C, ^{15}N]-EmrE/EmrE^{E14Q} (red, left panel) or [^{13}C, ^{15}N]-EmrE^{E14Q}/EmrE (red, right panel) in DMPC lipid bilayers bound to TPP at pH 5.0. Superscripts "A" or "B" refer to the corresponding monomer A or B, respectively.

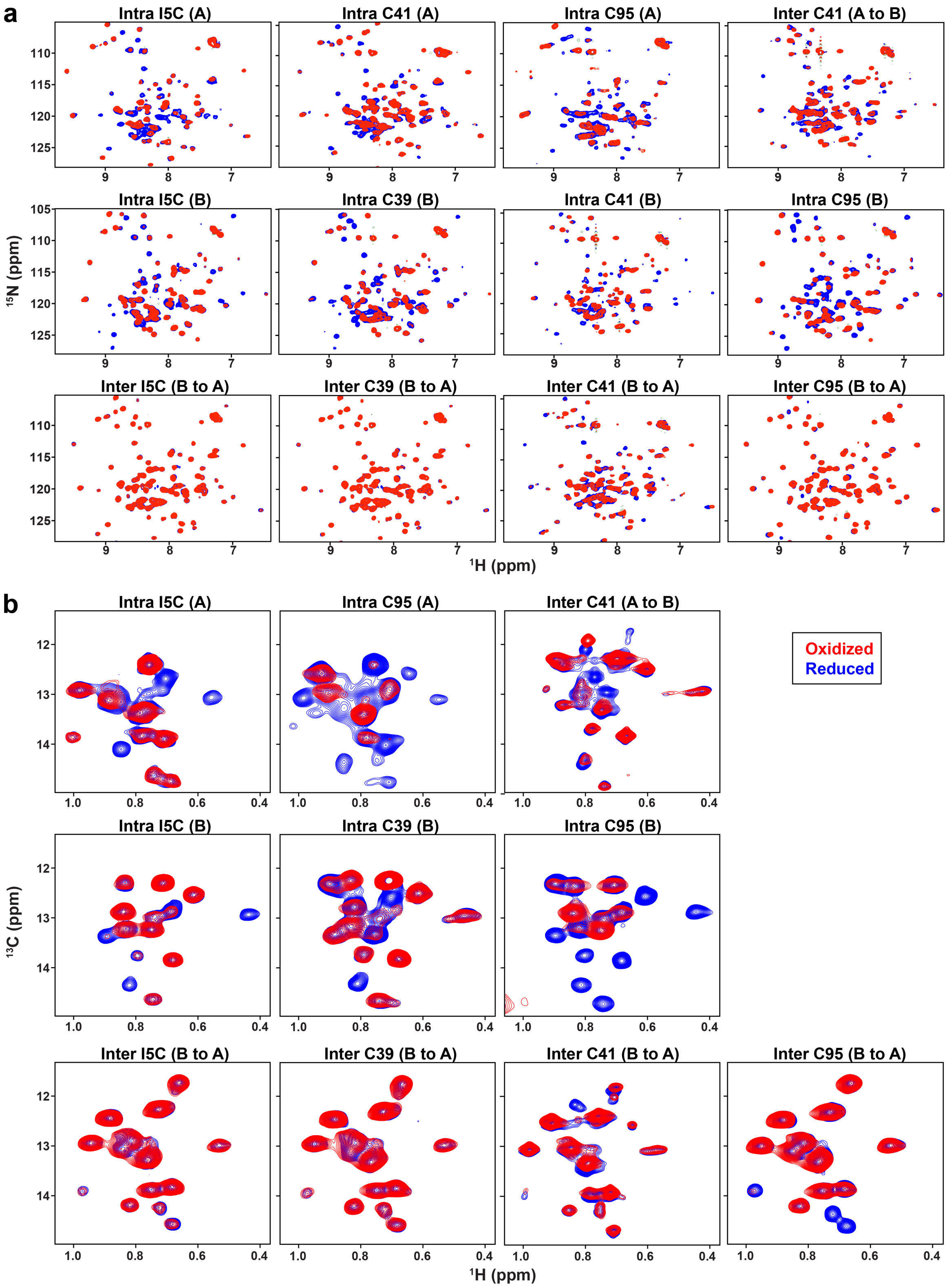

Supplementary Figure 2. Solution NMR PRE experiments of EmrE in the proton-bound form.

a, b. $^{1}H/^{15}N$ TROSY spectra (a) and $^{1}H/^{13}C$ HMQC spectra (b) of PRE samples of $EmrE^{L51I}$/EmrE heterodimers used to derive distance constraints for the proton-bound form. In each panel, the MTSL oxidized spectrum is displayed in red, while the reduced spectrum is displayed in blue. The sample nomenclature is as follows: "Intra" refers to intramolecular PRE constraints where MTSL is covalently attached at the indicated cysteine residue with the corresponding monomer displayed in parentheses. For example, "Intra I5C (A)" corresponds to $^{15}N/^{13}C$ labeled $EmrE^{L51I}$ with the MTSL tag at Cys5 and mixed with unlabeled EmrE. "Inter" refers to intermolecular constraints. For example, "Inter C41 (A to B)" corresponds to $^{15}N/^{13}C$ labeled EmrE (monomer B) mixed with MTSL labeled $EmrE^{L51I}$ at Cys41 (monomer A).

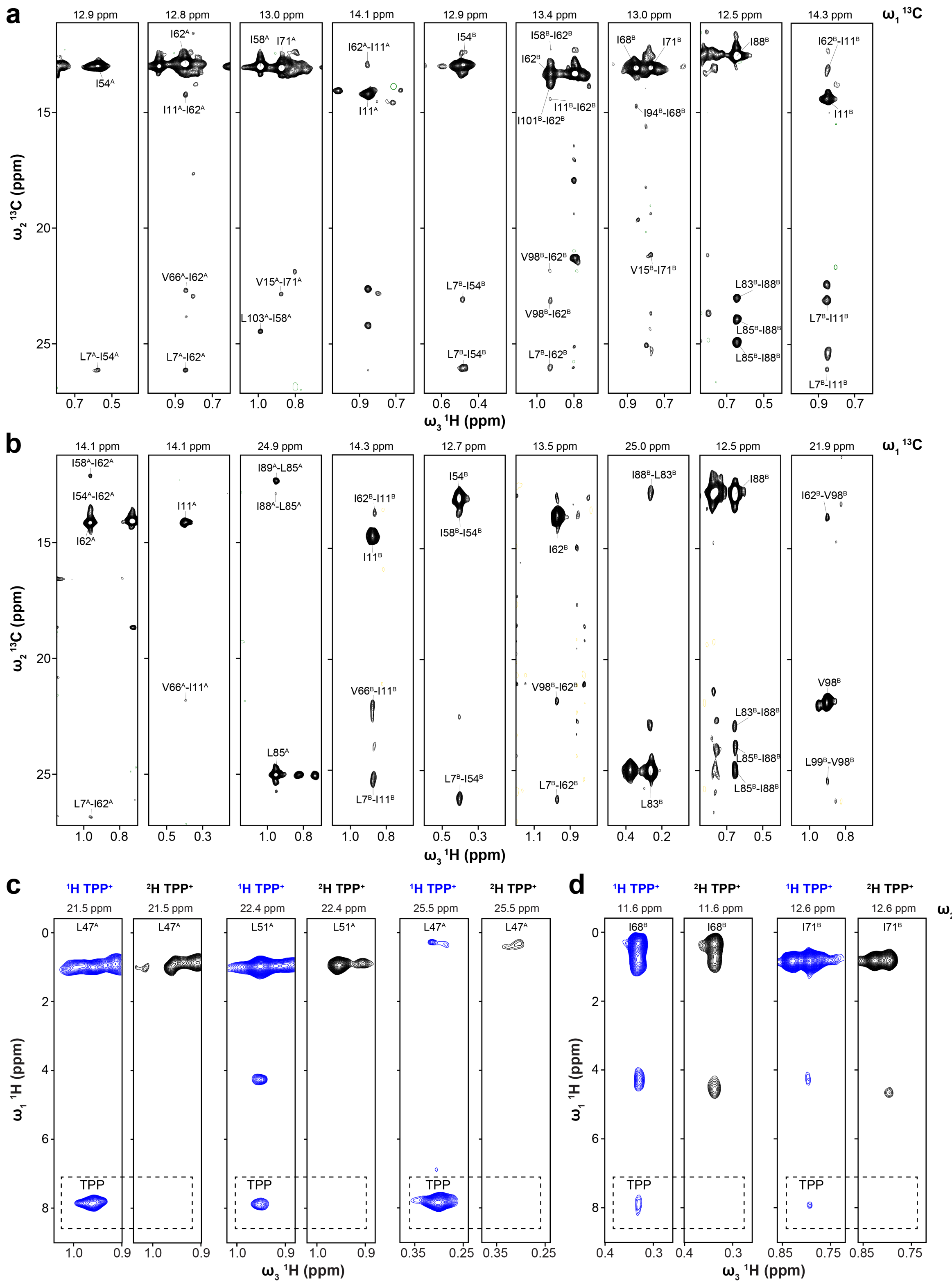

Supplementary Figure 3. Solution NMR NOE experiments of EmrE in the proton-bound and TPP-bound forms.

a, b. $^{1}H/^{13}C/^{13}C$ HMQC-NOESY-HMQC spectra of $EmrE^{L51I}$/EmrE heterodimers where either $EmrE^{L51I}$ (a) or EmrE (b) was isotopically enriched with $–^{13}CH_3$ methyl groups at isoleucine, leucine, and valine. Strips correspond to $^{1}H/^{13}C$ spectral slices at the indicated ^{13}C frequency in the indirect dimension. Labeled cross-peaks with “A” or “B” in the superscript indicate intramolecular NOEs within monomer A or B, respectively.

c, d. $^{1}H/^{1}H/^{13}C$ HSQC-NOESY spectra of EmrE/$EmrE^{E14Q}$ heterodimers bound to TPP at pH 5.8 where either EmrE (c) or $EmrE^{E14Q}$ (d) was isotopically enriched with $–^{13}CH_3$ methyl groups at isoleucine, leucine, and valine. Strips correspond to $^{1}H/^{1}H$ spectral slices at the indicated ^{13}C frequency in the indirect dimension. Labeled “TPP” cross-peaks indicate intermolecular NOEs between monomer A or B and TPP. Spectra in blue were performed with TPP at natural abundance, while control spectra in black were performed with perdeuterated TPP. Superscripts “A” or “B” for indicated residues refer to the corresponding monomer.

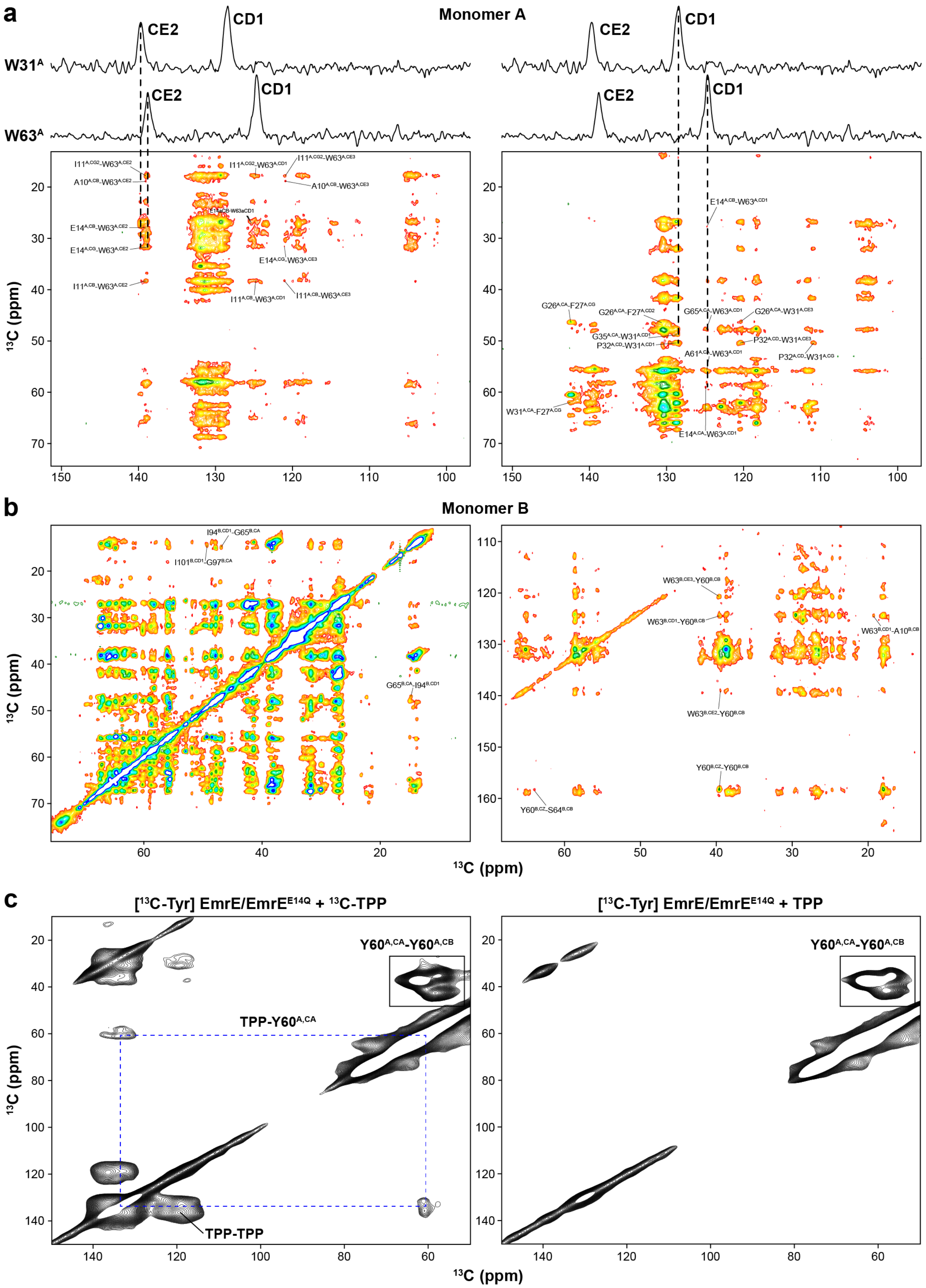

a
Monomer A
W31^{A}
W63^{A}
CE2
CD1
b
Monomer B
c
[^{13}C-Tyr] EmrE/EmrE^{E14Q} + ^{13}C-TPP
[^{13}C-Tyr] EmrE/EmrE^{E14Q} + TPP
Y60^{A,CA}-Y60^{A,CB}
TPP-Y60^{A,CA}
TPP-TPP
^{13}C (ppm)

Supplementary Figure 4. $^{13}C/^{13}C$ MAS solid-state NMR spectra of EmrE in the proton-bound and TPP-bound forms.

a, b. $^{13}C/^{13}C$ PDSD spectra of EmrE^{L51I}/EmrE heterodimers where either EmrE^{L51I} (a) or EmrE (b) was isotopically enriched with 1,3-^{13}C glycerol (left) or 2-^{13}C glycerol (right). Spectra in panel "a" were acquired with a 1 sec mixing time, while spectra in panel "b" were acquired with a 0.5 sec mixing time. Assigned cross-peaks are labeled in each of the panels, where superscripts "A" or "B" refer to the corresponding monomer. The 1D ^{13}C slices in panel "a" were extracted from the $^{13}C/^{15}N$ MAS correlation spectra recorded on the EmrE^{L51I}/EmrE heterodimer, where EmrE^{L51I} was isotopically labeled.

c. $^{13}C/^{13}C$ PDSD spectra with a 1 sec mixing time obtained using dynamic nuclear polarization at ~100 K. The sample was comprised of ^{13}C-Tyr ($^{13}C^{\alpha,\beta}$) labeled EmrE mixed with natural abundance EmrE^{E14Q} in the presence of TPP at pH 6.2. The spectrum on the left corresponds to addition of ^{13}C-labeled TPP while the spectrum on the right corresponds to addition of TPP at natural abundance. Cross-peaks highlighted by a dashed blue box show an intermolecular contact between TPP and Tyr60 of monomer A.

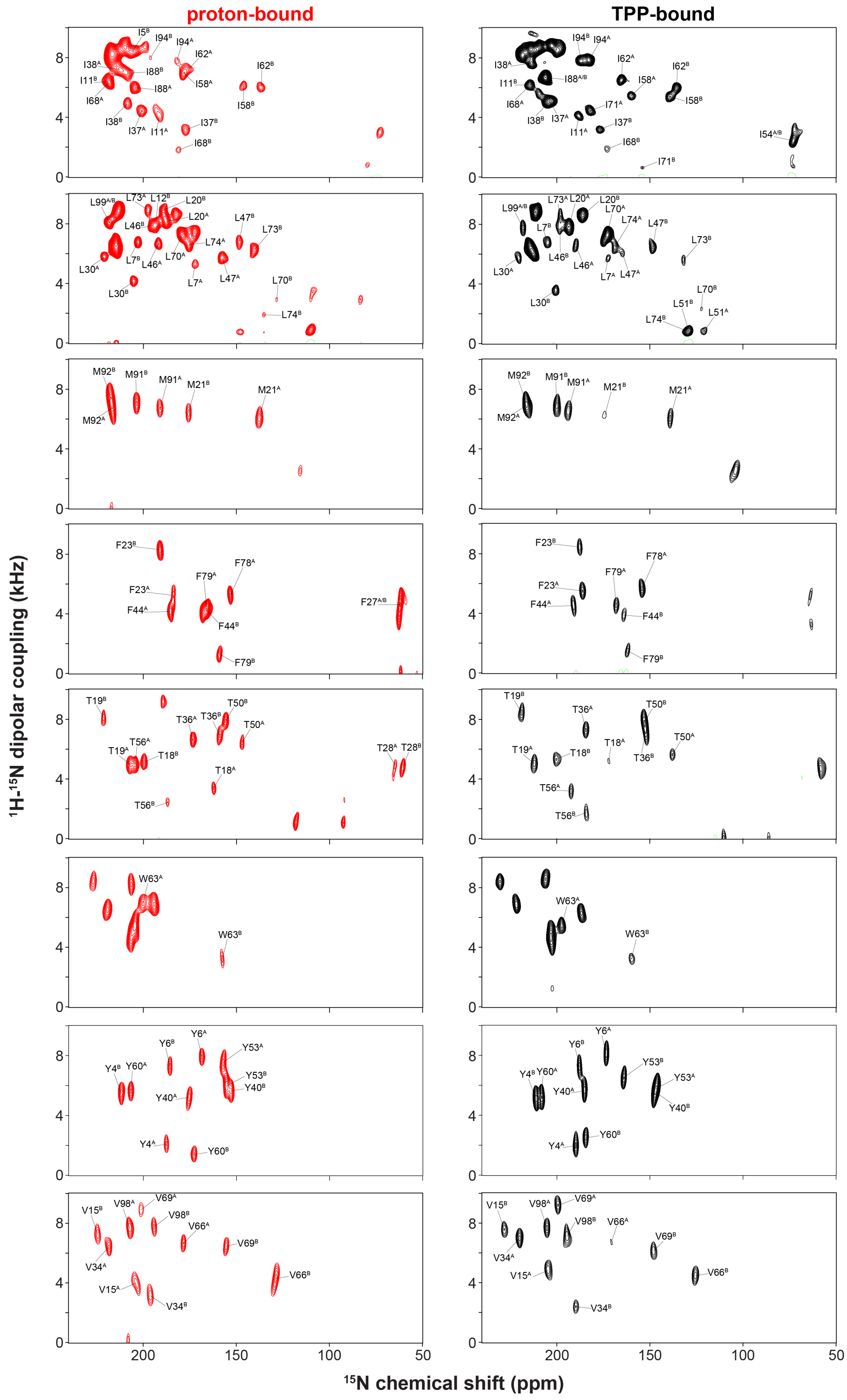

Supplementary Figure 5. PISEMA solid-state NMR spectra of EmrE in the proton-bound and TPP-bound forms.

PISEMA spectra acquired on wild-type EmrE in the proton-bound (left panels) and TPP-bound states (right panels) for several ^{15}N selectively labeled samples. From top to bottom, the

selective amino acid labeling is ^{15}N-Ile, ^{15}N-Leu, ^{15}N-Met, ^{15}N-Phe, ^{15}N-Thr, ^{15}N-Trp, ^{15}N-Tyr, and ^{15}N-Val. In each panel, the assignments are indicated in a monomer specific manner as indicated by "A" or "B" superscripts.

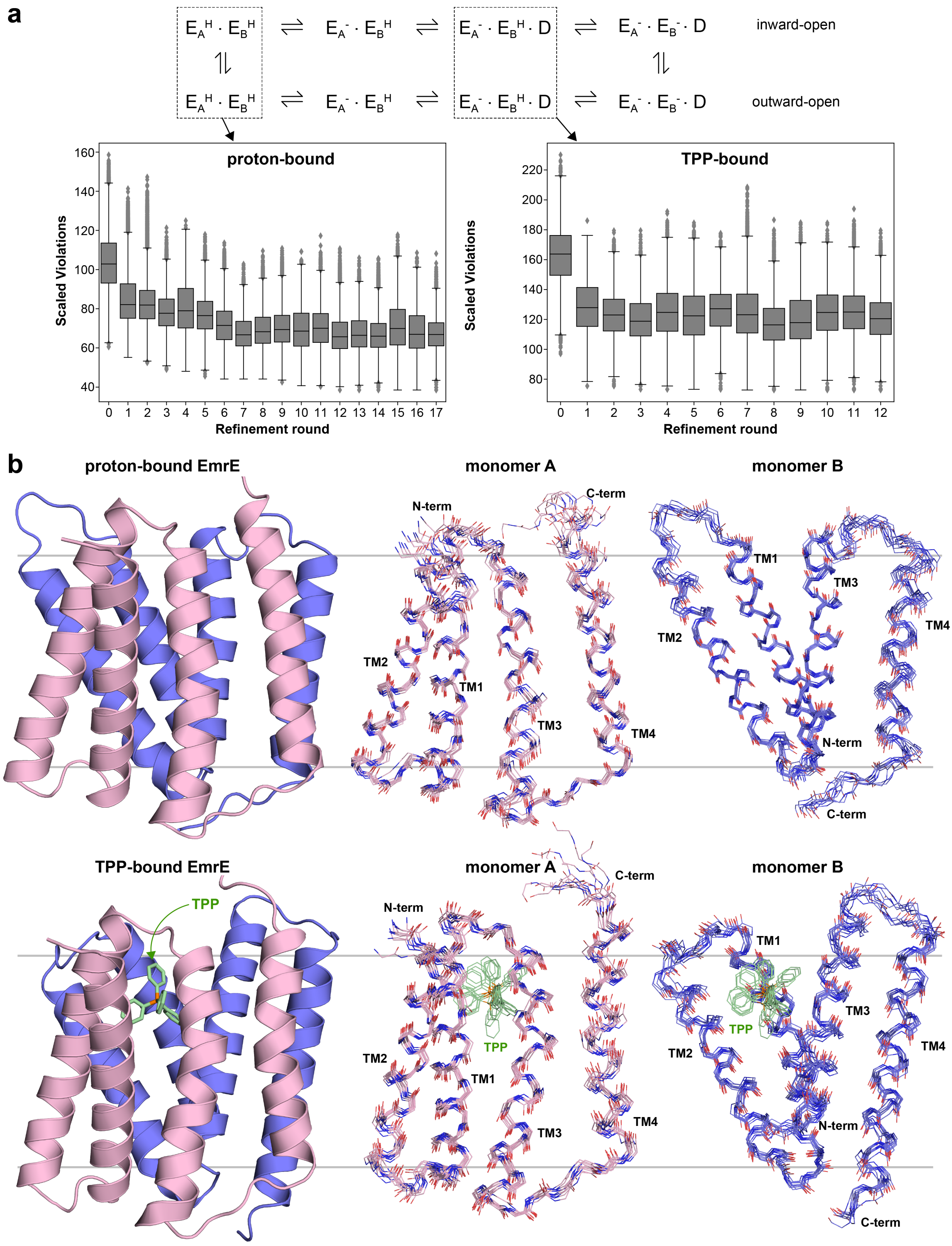

Supplementary Figure 6. EmrE structural ensemble of proton- and TPP-bound states.

a. Top: Transport cycle of EmrE where subscripts "A" or "B" correspond to the monomer in the structure, superscripts correspond to protonated ("H") or deprotonated ("–") states of

Glu14, and "D" corresponds to drug. The upper and lower states denote inward-open and outward-open conformations, respectively. The dotted boxes correspond to structures solved in this work. Since wild-type EmrE is a homodimer, structures solved can be viewed as cytoplasmic or periplasmic facing states. Bottom: Box and whisker plot of scaled violations calculated for each structure relative to experimental distance restraints after each MD simulation round in explicit lipid bilayers for proton-bound EmrE (left) and TPP-bound EmrE (right). The grey boxes correspond to the standard deviation, error bars are three times the standard deviation, and grey diamonds are all points outside the error bar range. Scaled violations are calculated as in Equation 1.

b. Left: Side views of the proton-bound EmrE^{L51I}/EmrE heterodimer structure (top) and the TPP-bound EmrE/EmrE^{E14Q} heterodimer structure (bottom) in a cartoon representation. Monomers A and B are displayed in pink and blue, respectively, and TPP is displayed in green. Middle, right: backbone overlay of the 10 lowest violated structures from MD simulations deposited in the PDB with the same view and coloring as in the left panels.

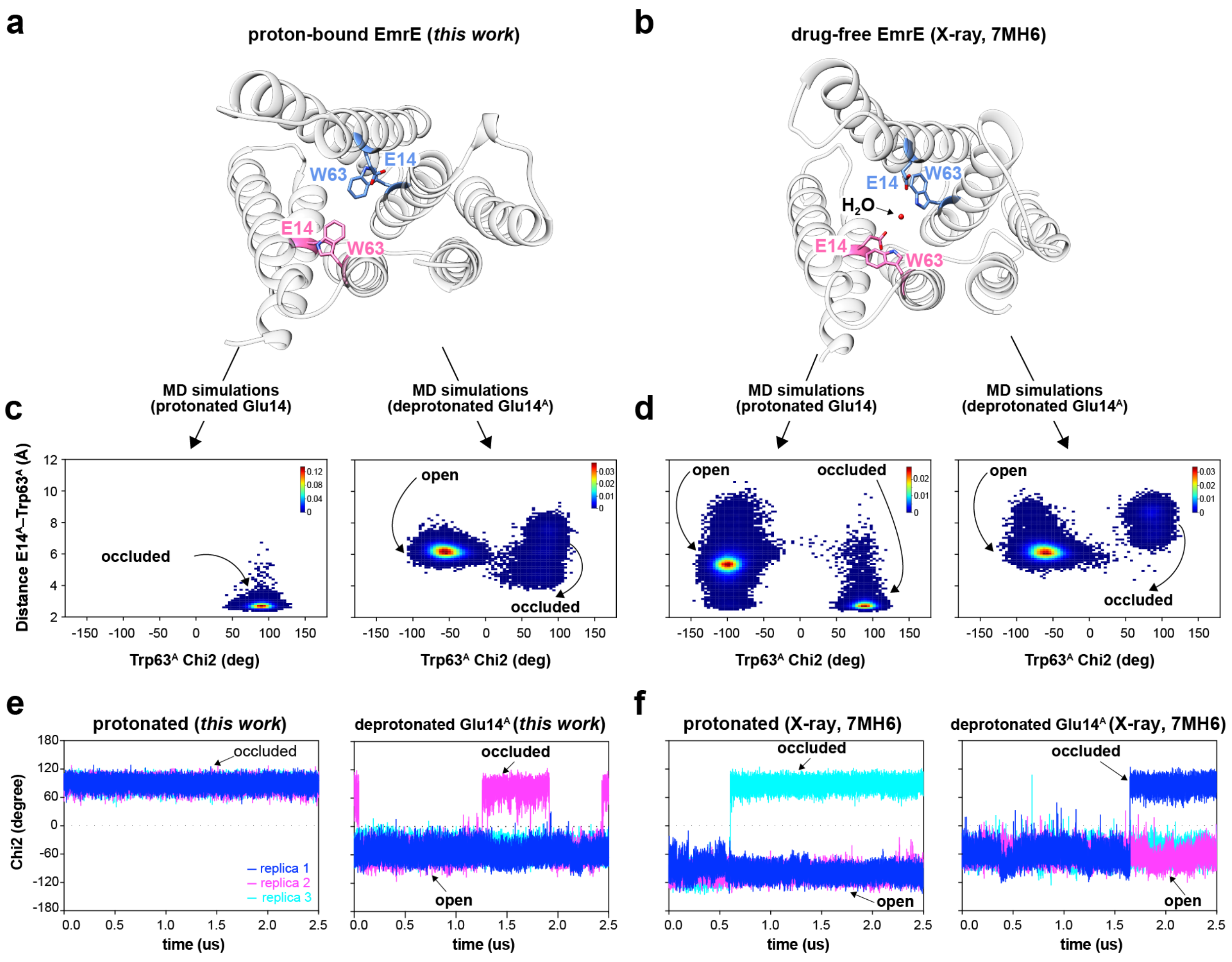

Supplementary Figure 7. Comparison of the proton-bound EmrE structure determined in this work with a drug-free X-ray structure and corresponding MD simulations.

a, b. Comparison of the proton-bound structure in this work (a) with a drug-free structure of EmrE determined using X-ray crystallography (b; PDB ID 7MH6). Residues of Glu14 and Trp63 are displayed in sticks and colored in pink for monomer A and in blue for monomer B. The red sphere in the X-ray structure shows a water molecule.

c, d. MD simulation analyses for proton-bound EmrE (left) or deprotonated EmrE at Glu14 of monomer A (right) starting from the proton-bound NMR structure (c) or the X-ray structure (d; PDB ID 7MH6). Heat map plots display the minimum distance between the side chain carboxyl oxygens of Glu14 of monomer A and the backbone carbonyl oxygen of Trp63 of monomer A versus the chi2 angle of Trp63 of monomer A.

e, f. Display of the chi2 angles as a function of the simulation time initiated from the NMR structure (e) or X-ray structure (f). The three replicate runs are displayed in different colors and are the same data as in panel (c, d).

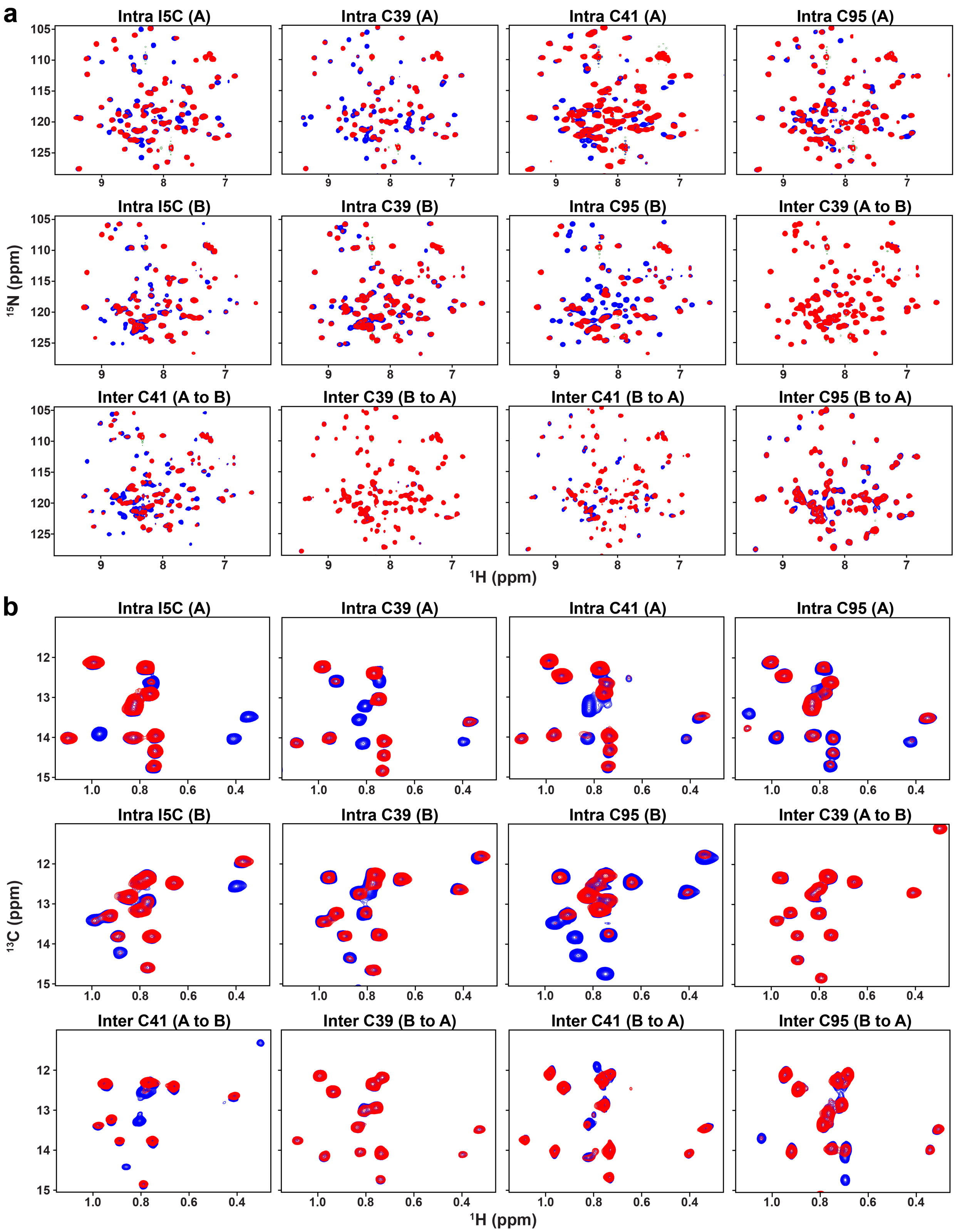

Supplementary Figure 8. Solution NMR PRE experiments of EmrE in the TPP-bound form.

a, b. $^{1}H/^{15}N$ TROSY spectra (a) and $^{1}H/^{13}C$ HMQC spectra (b) of PRE experiments on EmrE/EmrE^{E14Q} heterodimers bound to TPP. In each panel, the MTSL oxidized spectrum is displayed in red and the reduced spectrum is displayed in blue. The sample nomenclature is as follows: "Intra" refers to intramolecular PRE constraints where MTSL is covalently attached at the indicated cysteine residue with the corresponding monomer displayed in parentheses. For example, "Intra I5C (A)" corresponds to $^{15}N/^{13}C$ labeled EmrE with the MTSL tag at Cys5 and mixed with unlabeled EmrE^{E14Q}. "Inter" refers to intermolecular constraints. For example, "Inter C39 (A to B)" corresponds to $^{15}N/^{13}C$ labeled EmrE^{E14Q} (monomer B) mixed with MTSL labeled EmrE at Cys39 (monomer A).

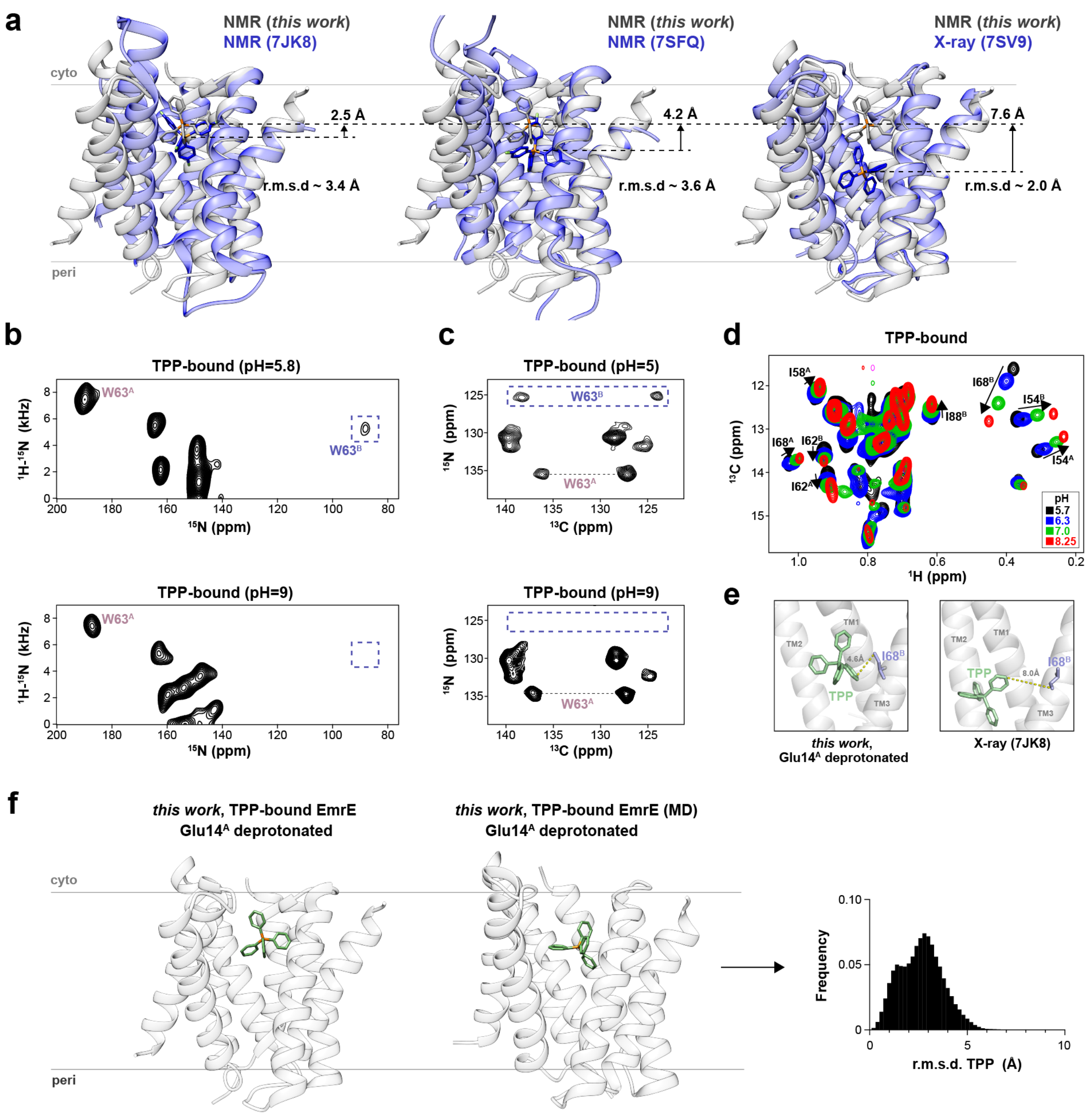

Supplementary Figure 9. Role of Glu14 deprotonation in TPP-bound EmrE.

a. Superimpositions of TPP-bound structure in this work (grey) to ones solved using solid-state NMR at pH 5.8 (left, blue; PDB ID 7JK8) or pH 8.0 (middle, blue; PDB ID 7SFQ) and X-ray crystallography at pH 7.25 (right, blue; PDB ID 7SV9). TPP is displayed in sticks and in the same color as EmrE.

b. PISEMA spectra of ^{15}N-indole tryptophan labeled EmrE bound to TPP at low (top) and high pH (bottom) values. Labeled signals of Trp63 for monomer A and B are colored in pink and blue, respectively. Superscripts "A" or "B" refer to the corresponding monomer.

c. MAS $^{13}C/^{15}N$ correlation spectra of the side chain tryptophan region for wild-type EmrE bound to TPP at low (top) and high pH (bottom) values. Labeled Trp63 signals of Trp63 for monomer A and B are colored and highlighted in rectangular boxes in pink and blue, respectively. Superscripts “A” or “B” refer to the corresponding monomer.

d. $^{1}H/^{13}C$ HMQC spectra of ^{13}C-Ile methyl labeled EmrE bound to TPP at the indicated pH values in DMPC/DHPC isotropic bicelles at 37 °C. Peak labels indicate the assigned isoleucine residue with monomer A or B displayed in the superscript.

e. Structural views of our NMR TPP-bound conformation of EmrE/EmrE^{E14Q} heterodimer (left) and the X-ray structure of EmrE in complex with TPP crystallized at pH 6.5 (right). TPP is colored in green with monomer B of EmrE displayed as a grey cartoon representation. The dashed yellow lines indicate the closest distance between the heavy atoms of Ile68 in monomer B and TPP (in Å).

f. Left, middle: our TPP-bound EmrE structure (left) and a representative snapshot from MD simulations starting from this structure (middle), where Glu14 of monomer A is deprotonated and Glu14 of monomer B is protonated. Right: r.m.s.d. of TPP for the MD simulation plotted as a frequency distribution and calculated from the phosphorus atom after aligning the protein backbone to the starting structure.

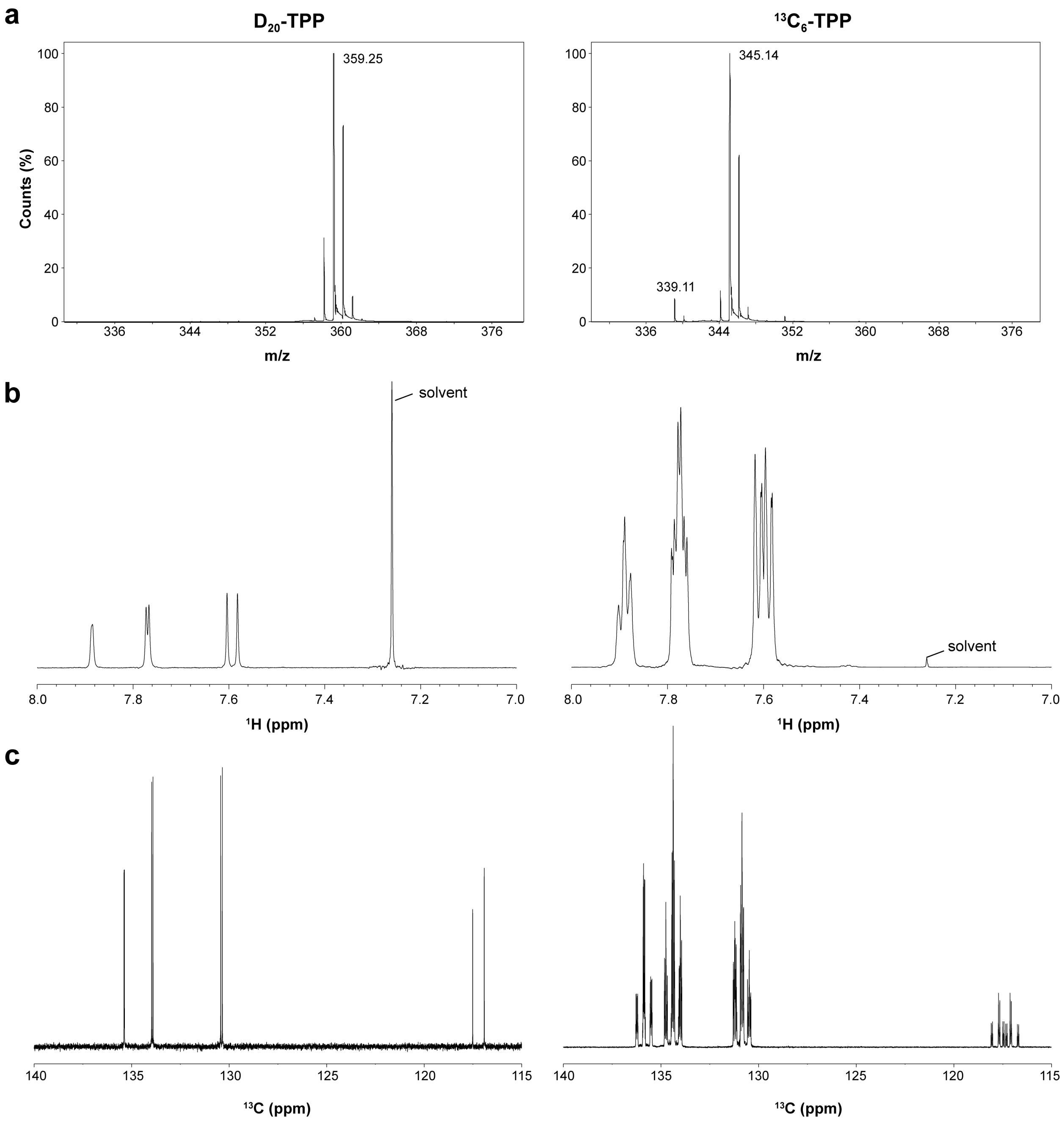

Supplementary Figure 10. Chemical characterization of isotopically enriched TPP compounds.

a. Mass spectrometry data for D_{20}-TPP (left) and $^{13}C_6$-TPP (right) with the major species m/z values displayed.

b, c. One-pulse ^1H NMR spectra with ^{13}C decoupling (b) and ^{13}C NMR spectra with ^1H decoupling (c) for D_{20}-TPP (left) and $^{13}C_6$-TPP (right). Note that the solvent peak is significantly more prominent for D_{20}-TPP due to the extent of deuterium incorporation.

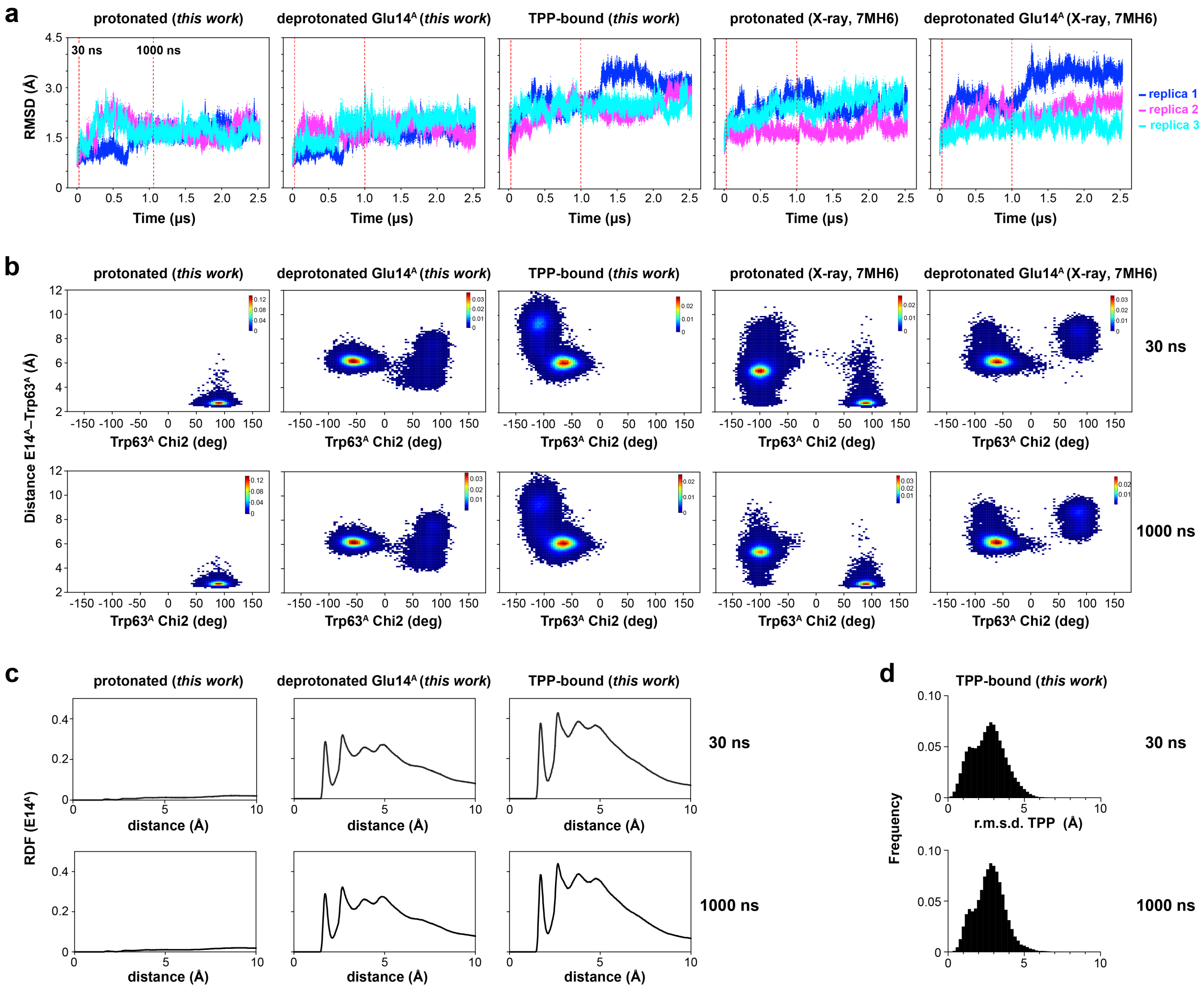

Supplementary Figure 11. Comparison of MD simulation analyses after 30 nsec and 1 μsec of equilibration.

a. Backbone r.m.s.d. of MD simulations initiated from the indicated structures of EmrE. The three replicates performed for each form of EmrE are displayed in the different colors. Analyses in subsequent panels were performed after 30 nsec or 1 μsec as indicated by the red dotted lines.

b. Heat map plots displaying the distance between the side chain carboxyl oxygen of Glu14^{A} and the backbone carbonyl oxygen of Trp63^{A} against the chi2 angle of Trp63^{A} (superscripts refer to monomer A) obtained from MD simulations initiated from the indicated structures of EmrE. Analyses were performed after 30 nsec (top row) or 1 μsec (bottom row).

c. Water radial distribution function (RDF) surrounding Glu14 of monomer A derived from MD simulations on proton-bound EmrE (left), EmrE deprotonated at Glu14 of monomer A (middle), and EmrE bound to TPP at low pH (right). Analyses were performed after 30 nsec (top row) or 1 μsec (bottom row).

d. r.m.s.d. of TPP for the MD simulation plotted as a frequency distribution and calculated from the phosphorus atom after aligning the protein backbone to the starting structure. Analyses were performed after 30 nsec (top row) or 1 μsec (bottom row).

Supplementary Table 1. NMR and refinement statistics for protein structures

	$EmrE^{L51I}$/EmrE (proton-bound)	EmrE/$EmrE^{E14Q}$ (TPP-bound)
**PDB**	8UWU	8UOZ
**NMR constraints**		
Distance constraints (DARR, NOE, PRE)		
Total restraints	948	1236
Intra-residue	1	1
Inter-residue		
Sequential ($\|i-j\| = 1$)	26	42
Medium-range ($\|i-j\| \leq 4$)	51	134
Long-range ($\|i-j\| \geq 5$)	477	569
Intermolecular (intermonomer)	393	483
Intermolecular (to TPP)	–	7
Total backbone dihedral angle restraints		
ϕ	90	97
ψ	90	97
Angular PISEMA restraints		
^{15}N chemical shifts	87	88
^{1}H-^{15}N dipolar couplings	87	88
**Structure statistics**		
Violations (mean and s.d.)*		
Distance constraints (Å)	1.48 ± 1.41	1.47 ± 1.31
Dihedral angle constraints (°)	39.5 ± 51.5	21.6 ± 33.0
Max. dihedral angle violation (°)	159.0	158.9
Max. distance constraint violation (Å)	8.55	6.85
Average pairwise r.m.s. deviation** (Å)		
Heavy	1.64	1.78
Backbone	1.03	1.10

* Structural ensembles were derived using unrestrained MD simulations in the absence of NMR constraints. The structures of the ensemble correspond to snapshots selected from the simulation that were in best agreement with the experimental data. Note that 95.5% to 96.2% of the distance restraints were satisfied for the proton-bound EmrE ensemble and 94.2% to 94.9% for the TPP-bound ensemble. Most violations likely reflect an imprecise location of the MTSL tag in PRE experiments (i.e., constraints implemented from the sulfur of cysteine, the CG1 of isoleucine, or the CG of leucine).

** Pairwise r.m.s. deviation was calculated among 10 refined structures in each ensemble. The ensembles were determined using unrestrained all-atom MD simulations in DMPC lipid bilayers by selecting snapshots with the fewest violations of experimental constraints.

Supplementary Table 2. MD simulation setup details

	Proton-bound EmrE (*this work*)	Deprotonated at $E14^A$ EmrE (*this work*)	TPP-bound EmrE (*this work*)	Proton-bound EmrE (X-ray)	Deprotonated at $E14^A$ EmrE (X-ray)
**Starting PDB**	8UWU	8UWU	8UOZ	7MH6	7MH6
**Simulation box dimensions (Å)**	80 x 69 x 92	80 x 69 x 92	80 x 69 x 94	80 x 70 x 88	80 x 70 x 88
**Total atoms**	46,669	46,646	47,285	44,553	44,722
**Total waters**	9,057	9,050	9,377	8,349	8,406
**Salt concentration**	20 mM NaCl	20 mM NaCl	20 mM NaCl	20 mM NaCl	20 mM NaCl
**Total lipids**	136 DMPC	136 DMPC	133 DMPC	138 DMPC	138 DMPC

References

1. Thomas, N. E. *et al.* The C terminus of the bacterial multidrug transporter EmrE couples drug binding to proton release. *J Biol Chem* **293**, 19137-19147 (2018). https://doi.org/10.1074/jbc.RA118.005430
2. Shcherbakov, A. A., Spreacker, P. J., Dregni, A. J., Henzler-Wildman, K. A. & Hong, M. High-pH structure of EmrE reveals the mechanism of proton-coupled substrate transport. *Nat Commun* **13**, 991 (2022). https://doi.org/10.1038/s41467-022-28556-6
