# Supplementary material for: Dynamics underlie the drug recognition mechanism by the efflux transporter EmrE
Source: Nat Commun. 2024 May 28;15:4537. doi: 10.1038/s41467-024-48803-2 (PMC11133458; doi:10.1038/s41467-024-48803-2)
Supplement: Supplementary file 3 — Reporting Summary [file 41467_2024_48803_MOESM3_ESM.pdf]

nature research

Corresponding author(s): Nathaniel J. Traaseth

Last updated by author(s): Apr 29, 2024

Reporting Summary

Nature Research wishes to improve the reproducibility of the work that we publish. This form provides structure for consistency and transparency in reporting. For further information on Nature Research policies, see our Editorial Policies and the Editorial Policy Checklist.

Statistics

For all statistical analyses, confirm that the following items are present in the figure legend, table legend, main text, or Methods section.

n/a	Confirmed	
☐	☒	The exact sample size (n) for each experimental group/condition, given as a discrete number and unit of measurement
☐	☒	A statement on whether measurements were taken from distinct samples or whether the same sample was measured repeatedly
☒	☐	The statistical test(s) used AND whether they are one- or two-sided<br>*Only common tests should be described solely by name; describe more complex techniques in the Methods section.*
☒	☐	A description of all covariates tested
☒	☐	A description of any assumptions or corrections, such as tests of normality and adjustment for multiple comparisons
☐	☒	A full description of the statistical parameters including central tendency (e.g. means) or other basic estimates (e.g. regression coefficient) AND variation (e.g. standard deviation) or associated estimates of uncertainty (e.g. confidence intervals)
☒	☐	For null hypothesis testing, the test statistic (e.g. F, t, r) with confidence intervals, effect sizes, degrees of freedom and P value noted<br>*Give P values as exact values whenever suitable.*
☒	☐	For Bayesian analysis, information on the choice of priors and Markov chain Monte Carlo settings
☒	☐	For hierarchical and complex designs, identification of the appropriate level for tests and full reporting of outcomes
☒	☐	Estimates of effect sizes (e.g. Cohen's d, Pearson's r), indicating how they were calculated

Our web collection on statistics for biologists contains articles on many of the points above.

Software and code

Policy information about availability of computer code

Data collection	ITC: NanoITCRun 3.12.5<br>Tryptophan Fluorescence: SoftMax Pro 7.1.2<br>Growth inhibition: Bioscreen C Pro<br>NMR Spectroscopy: TopSpin 3.5 pl 7 and VnmrJ 3.1 rev. A<br>MD simulations: GROMACS 2020.4
Data analysis	ITC: TA Instruments NanoAnalyze 3.12.5<br>Growth inhibition: Prism software version 10.0.3 (217)<br>NMR spectroscopy: NMRPipe Version 8.1 and Sparky 3.115<br>Structure Determination: Xplor-NIH 2.52, GROMACS 2020.4, and python 3.8.5 with mdtraj 1.9.4 and mdanalysis 1.0.0.<br>MD simulations: GROMACS 2020.4 and VMD 1.9.3.

For manuscripts utilizing custom algorithms or software that are central to the research but not yet described in published literature, software must be made available to editors and reviewers. We strongly encourage code deposition in a community repository (e.g. GitHub). See the Nature Research guidelines for submitting code & software for further information.

Data

Policy information about availability of data

All manuscripts must include a data availability statement. This statement should provide the following information, where applicable:

- Accession codes, unique identifiers, or web links for publicly available datasets
- A list of figures that have associated raw data
- A description of any restrictions on data availability

The datasets generated during and/or analyzed during the current study are deposited in the Protein Data Bank and Biological Magnetic Resonance Bank for proton-bound EmrE (PDB ID: 8UWU [https://doi.org/10.2210/pdb8UWU/pdb]; BMRB ID: 31125) and TPP-bound EmrE (PDB ID: 8UOZ [https://doi.org/10.2210/pdb8UOZ/pdb]; BMRB ID: 31121). MD simulation input and coordinates of starting and ending structure are publicly available using the link: https://doi.org/10.5281/zenodo.10999382. The previously published structures were referred in this paper: 7MH6 [https://doi.org/10.2210/pdb7MH6/pdb], 7JK8 [https://doi.org/10.2210/pdb7JK8/pdb], 7SFQ [https://doi.org/10.2210/pdb7SFQ/pdb], 7SV9 [https://doi.org/10.2210/pdb7SV9/pdb] and 3B5D [https://doi.org/10.2210/pdb3B5D/pdb]. The source data underlying Figures 3e, 4a, 4b, 4d, 4e, 4g,4h, and Supplementary Figure 1a, 1c, 6a, 7d, 7e, 7f, 9f are provided as a Source Data file.

Field-specific reporting

Please select the one below that is the best fit for your research. If you are not sure, read the appropriate sections before making your selection.

☒ Life sciences ☐ Behavioural & social sciences ☐ Ecological, evolutionary & environmental sciences

For a reference copy of the document with all sections, see nature.com/documents/nr-reporting-summary-flat.pdf

Life sciences study design

All studies must disclose on these points even when the disclosure is negative.

Sample size	Binding and growth inhibition assays were performed in duplicate or more. The sample sizes are listed for each sample in the respective Methods section and/or in the figure legends.
Data exclusions	No data were excluded from the analyses.
Replication	Functional assays were replicated in independent experiments. Structures determined were tested in multiple molecular dynamics runs and replicate the overall stability of the structural ensembles reported.
Randomization	Randomization of datasets was not applicable for this manuscript.
Blinding	Blinding of datasets was not applicable for this manuscript

Reporting for specific materials, systems and methods

We require information from authors about some types of materials, experimental systems and methods used in many studies. Here, indicate whether each material, system or method listed is relevant to your study. If you are not sure if a list item applies to your research, read the appropriate section before selecting a response.

Materials & experimental systems

n/a	Involved in the study
☒	☐ Antibodies
☒	☐ Eukaryotic cell lines
☒	☐ Palaeontology and archaeology
☒	☐ Animals and other organisms
☒	☐ Human research participants
☒	☐ Clinical data
☒	☐ Dual use research of concern

Methods

n/a	Involved in the study
☒	☐ ChIP-seq
☒	☐ Flow cytometry
☒	☐ MRI-based neuroimaging
